# Spontaneous long-range calcium waves in developing butterfly wings

**DOI:** 10.1186/s12861-015-0067-8

**Published:** 2015-03-25

**Authors:** Yoshikazu Ohno, Joji M Otaki

**Affiliations:** The BCPH Unit of Molecular Physiology, Department of Chemistry, Biology and Marine Science, Faculty of Science, University of the Ryukyus, Nishihara, Okinawa 903-0213 Japan

**Keywords:** Butterfly wing, Calcium wave, Color pattern development, Eyespot, *In vivo* imaging, *Junonia orithya*, Long-distance signaling, Physical damage, Thapsigargin

## Abstract

**Background:**

Butterfly wing color patterns emerge as the result of a regular arrangement of scales produced by epithelial scale cells at the pupal stage. These color patterns and scale arrangements are coordinated throughout the wing. However, the mechanism by which the development of scale cells is controlled across the entire wing remains elusive. In the present study, we used pupal wings of the blue pansy butterfly, *Junonia orithya*, which has distinct eyespots, to examine the possible involvement of Ca^2+^ waves in wing development.

**Results:**

Here, we demonstrate that the developing pupal wing tissue of the blue pansy butterfly displayed spontaneous low-frequency Ca^2+^ waves *in vivo* that propagated slowly over long distances. Some waves appeared to be released from the immediate peripheries of the prospective eyespot and discal spot, though it was often difficult to identify the specific origins of these waves. Physical damage, which is known to induce ectopic eyespots, led to the radiation of Ca^2+^ waves from the immediate periphery of the damaged site. Thapsigargin, which is a specific inhibitor of Ca^2+^-ATPases in the endoplasmic reticulum, induced an acute increase in cytoplasmic Ca^2+^ levels and halted the spontaneous Ca^2+^ waves. Additionally, thapsigargin-treated wings showed incomplete scale development as well as other scale and color pattern abnormalities.

**Conclusions:**

We identified a novel form of Ca^2+^ waves, spontaneous low-frequency slow waves, which travel over exceptionally long distances. Our results suggest that spontaneous Ca^2+^ waves play a critical role in the coordinated development of scale arrangements and possibly in color pattern formation in butterflies.

**Electronic supplementary material:**

The online version of this article (doi:10.1186/s12861-015-0067-8) contains supplementary material, which is available to authorized users.

## Background

In biological systems, cellular communication is mediated by various types of molecules, including signaling proteins. However, some inorganic ions, such as Na^+^, K^+^, and Ca^2+^, are known to be involved in important signaling processes, especially in tissues and organs that are composed of electrically excitable cells. The role of ions as signaling molecules is possible partially because their distribution and trafficking in cells, tissues and organs are tightly regulated by membranous structures. Among these signaling ions, calcium ions (Ca^2+^) are known to contribute to numerous cellular processes in both excitable and non-excitable cells during development and differentiation [1-3]. For example, in the central nervous system, glial Ca^2+^ signals play important roles in various physiological processes, including cellular proliferation and the coordination of neuronal metabolism [4-6]. Developmental and physiological roles of Ca^2+^ waves have also been well documented in the retina [7,8], the skeletal muscles [9], and the intact liver [10,11]. Spontaneous Ca^2+^ waves coordinate morphogenesis in zebrafish [12,13], and long-distance cellular communication through intercellular Ca^2+^ waves has also been reported in the slime mold [14]. In insects, Ca^2+^ waves have been well studied in the blowfly salivary gland [15]; however, the developmental roles of Ca^2+^ waves in insect tissues have not been elucidated [16]. We therefore examined these processes using pupal wing tissues of butterflies.

Butterfly wings harbor unique color patterns that are constructed by regularly arranged scales on the surface of the wing, akin to mosaic roof tiles. At the microscopic level, each scale has a single color that is produced by a single epithelial scale cell during the pupal stage [17]. At the macroscopic level, the color pattern of a wing is the result of a collection of color pattern elements [17]. The diverse color patterns of butterflies are due to a transformation of the nymphalid groundplan [17-20], which is a simple scheme of the manner by which color pattern elements are positioned against a plain background. One representative color pattern element is an eyespot (also called a border ocellus). The center of the prospective eyespot area functions as an organizing center from which a putative morphogenic signal that determines color patterns is believed to be released to the surrounding tissue [21-23]. Many putative genes that may be involved in this process are expressed in the prospective eyespot area [24-27], yet their functions remain elusive.

Although the nature of the morphogenic signals is not known, the color pattern determination process has been explained using a concentration gradient model for positional information [21-24]. This model has been applied to an ideally symmetric eyespot in the model butterfly *Bicyclus anynana* [21], but the actual eyespot structures of nymphalid butterflies are too diverse in shape to be explained by a simple gradient model [28]. Furthermore, damage-induced color pattern changes in *Junonia almana*, which have been examined at relatively high resolution, are not explainable by a simple concentration gradient model [29]. Such damage experiments have demonstrated that the inner and outer black rings of a single eyespot respond to damage independently, that damage near an eyespot can change the shape and size of a nearby eyespot, that damage can induce an orange background region inside of a black ring, and that when one eyespot is reduced in size due to damage, an adjacent eyespot increases in size [29]. Considering the diverse eyespots widely observed in nymphalid butterflies and the results of the damage experiments, an induction model has been proposed, in which morphogenic signals are not static concentration gradients but are instead generated as serial pulses or waves from an organizing center [30,31].

Over the course of butterfly evolution, repeated wing-wide systematic changes in the position, size, and shape of color pattern elements in the nymphalid groundplan may have occurred. Similar global changes in these elements can be induced by temperature shock or chemical injection [32-36], demonstrating that the fate of differentiating immature scale cells can be modified by environmental factors. It has been proposed that wing-wide signals, such as ecdysteroids and cold-shock hormones (CSHs), coordinate the overall color patterns of wings [33-35,37-39]. A global signal is also implicated by the positional dependence of scale size and shape [40,41]. Although this global signal may not be identical to morphogenic signals from organizing centers, we hypothesized that a long-range signal that travels across the wing plays an important role in the development of scales and epithelial scale cells and hence the color patterns of butterfly wings. We speculated that Ca^2+^ waves are the most likely to be involved in the mechanism underlying this function.

**Figure 1:**
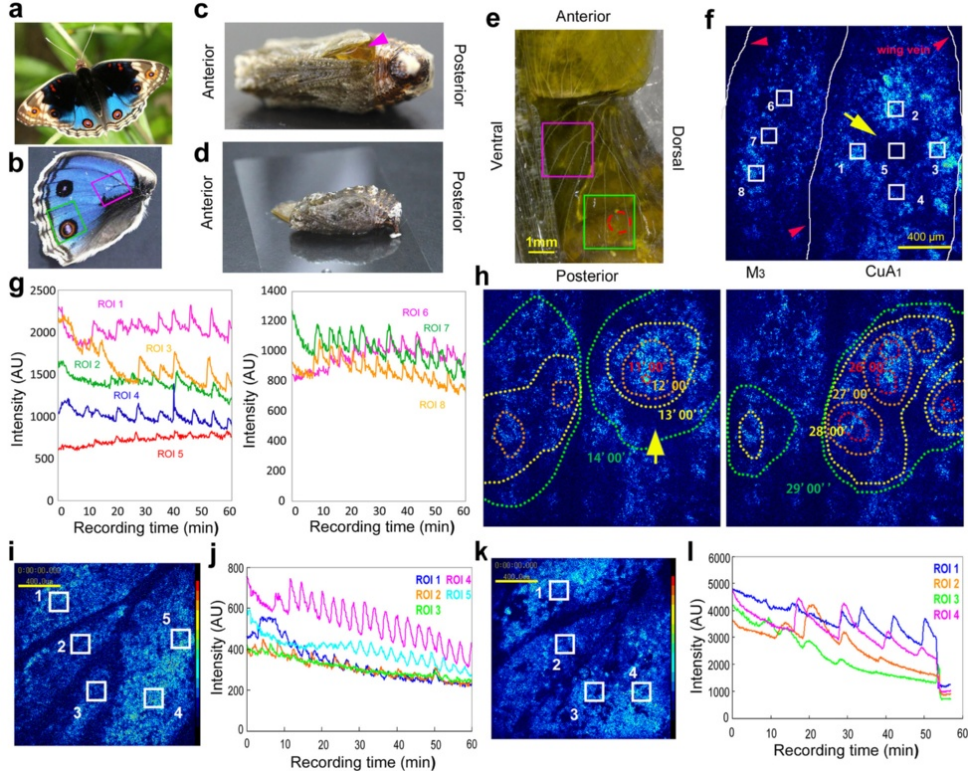
**Spontaneous Ca** ^**2+**^ **waves in pupal wing tissue. (a)** An adult individual of *Junonia orithya*. **(b)** Dorsal hindwing of *J. orithya*. The two boxed areas (the basal region in the red box and the border region in the green box) correspond to the areas of analysis in the pupal wing. **(c)** The loading of a chemical solution (the pink arrow) into the pupal wing tissue via the sandwich method. **(d, e)** The pupal hindwing attached to a piece of cover glass at approximately 1 h after surgery. The green rectangular area (the border region) corresponds to the area shown in **f** and **h**. The pink rectangular area (the basal region) corresponds to the area shown in **i** and **k**. The red broken circle corresponds to the prospective eyespot area. The scale bar represents 1 mm. **(f)** Ca^2+^ signals in the M_3_ and CuA_1_ compartments (the border region). ROIs 1–8 were examined for intensity changes in **g**. The yellow arrow indicates the prospective eyespot area, which is darker than its surroundings. The red arrowheads indicate the wing veins. The scale bar represents 400 μm. See also Additional file 1: Movie S1. **(g)** Fluorescence intensity changes in 8 ROIs in arbitrary units (AUs) over time. **(h)** Propagating Ca^2+^ signals around the prospective eyespot area as indicated by the yellow arrow. The right and left panels (and **f**) show a single identical visual field with superimposed propagating signals. A shape of a wave at a given time point (in min) is depicted by a dotted circle. **(i)** Ca^2+^ signals around the discal cell (i.e., the basal region). Five ROIs are defined. The scale bar represents 400 μm. See also Additional files 2, 3: Movies S2, S3. **(j)** Fluorescence intensity changes in the 5 ROIs defined in **i** over time. **(k)** Another example of Ca^2+^ signals around the discal cell. The scale bar represents 400 μm. **(l)** Fluorescence intensity changes in the 4 ROIs defined in **h** over time.

In this study, we investigated the potential involvement of Ca^2+^ waves in butterfly wing development using the blue pansy butterfly *Junonia orithya*, which has large eyespots on its wings (Figure 1a, b). We have previously established a system that enables us to directly observe developing wing tissues *in vivo* until eclosion without any detectable disturbing effects and to load chemicals into the developing wing [40,42,43] (Figure 1c, d). Using this system, we have characterized the manner by which pupal wing tissue develops over time *in vivo* [43]. Because this method is very simple and non-invasive, only involving wing displacement, the possibility of introducing artifacts is minimal (see Methods).

In the present study, we discovered Ca^2+^ waves in the developing wing tissues *in vivo*. Our results suggest that spontaneous and damage-associated Ca^2+^ waves that propagate slowly over long distances may play roles in the development and differentiation of pupal epithelial scale cells.

## Results

### Spontaneous long-distance Ca^2+^ waves across a wing

To observe calcium dynamics in an intact pupal hindwing, we used Fluo-4 AM or Fluo-8 AM, which are commonly used Ca^2+^ indicators. The indicators were successfully loaded into the wing tissues after surgery (Figure 1c-e). We first focused on the M_1_ and CuA_1_ compartments, here called the border region of the pupal wing tissue. The CuA_1_ compartment (but not the M_1_ compartment) contained the prospective eyespot area. However, the prospective eyespot area was resistant to the indicators (Figure 1f), as described previously [43]. This resistance was most likely because of the thick cuticle and dented structure of the eyespot area [43].

In the non-eyespot areas of the M_3_ and CuA_1_ compartments, we observed many spontaneous intercellular Ca^2+^ waves (i.e., fluorescence intensity changes, reflecting changes in the intracellular calcium concentration) that traveled slowly over long distances (*n* = 4; *n* designates the number of individuals examined hereafter) (Figure 1f, g). Several Ca^2+^ waves were observed simultaneously in various locations within a single visual field. When the Ca^2+^ waves were physically close to each other, they appeared to collide, reducing in velocity and amplitude. We detected periodic fluctuations in fluorescence intensity in defined regions of interest (ROIs) at approximately 5–10 min intervals (Figure 1g).

The positional origins of many of the Ca^2+^ waves were too diffuse to identify, which is partially because several waves crossed the visual field simultaneously and because our visual field was not large enough to pinpoint the origins of these waves. However, some Ca^2+^ waves appeared to propagate from the immediate periphery of the prospective eyespot in the 4 individuals examined (Figure 1h; Additional file 1: Movie S1). Although the prospective eyespot itself did not stain well as mentioned above, radially expanding rings that were observed from the immediate periphery of the prospective eyespot suggest that the organizing center may be able to emit Ca^2+^ waves.

Similarly, we next focused on the compartments near the prospective discal spot, here called the basal region of the pupal wing tissue. We detected several Ca^2+^ waves that appeared to originate from the basal compartments close to the prospective discal spot at intervals of approximately 5–10 min (*n* = 61) (Figure 1i-l; Additional file 2: Movie S2). It is worth noting that the discal spot is likely the organizing center of the central symmetry system [17-20]. We noticed that different individuals exhibited different wave patterns from similar locations (Figure 1i-l). Interestingly, complex wave behavior, including spiral or vortex movement, was observed in 4 of the 61 individuals examined (Additional file 3: Movie S3).

### Traveling distance and velocity of Ca^2+^ waves

**Figure 2:**
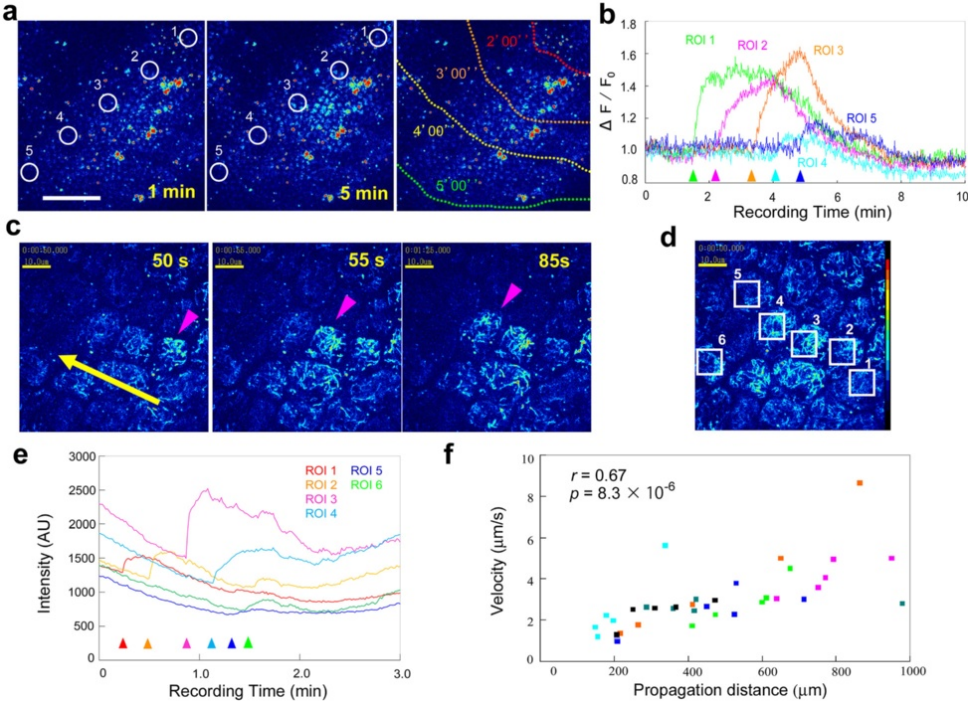
**Traveling Ca** ^**2+**^ **waves. (a)** Time course of fluorescence intensity changes. Shown are 1 min (left panel) and 5 min (middle panel) post-recording. ROIs 1–5 were set at regular intervals for analysis, as shown in **b**. The wave fronts traveled from the upper right to the lower left, as shown in the right panel (in min). The scale bar represents 100 μm. See also Additional file 4: Movie S4. **(b)** Intensity changes in the 5 ROIs defined in **a**. The arrowheads indicate sudden increases in intensity, i.e., wave fronts. **(c)** Time course of fluorescence intensity changes at the cellular level. The intensely highlighted structures are probably mitochondria. The yellow arrow indicates the direction of travel. The pink arrowheads indicate the wave-front cells. Ca^2+^ signals are elevated a few cells at a time. The scale bar represents 10 μm. See also Additional file 5: Movie S5. **(d)** The same visual field as **c**, in which 6 ROIs are defined to examine the intensity changes in **e**. The scale bar represents 10 μm. **(e)** Fluorescence intensity changes in the 6 ROIs defined in **d**. The wave fronts are indicated by arrowheads. **(f)** Scatter plot of the propagation distances and velocities of Ca^2+^ waves. Each dot represents a single wave, and the different colors represent different individuals. The Pearson correlation coefficient *r* and its associated *p*-value are shown.

To determine the manner by which the waves travel long distances, we evaluated a representative case from the basal area at which a Ca^2+^ wave crossed a visual field diagonally from the upper right side to the lower left side over a period of 5 min (Figure 2a, b; Additional file 4: Movie S4). We set 5 ROIs containing a few cells in the direction of wave propagation at regular intervals (Figure 2a). The Ca^2+^ wave appeared to decrease in intensity as it traveled (Figure 2b).

Another Ca^2+^ wave traveling diagonally from the lower right side to the upper left side in a visual field was also captured at the cellular level (Figure 2c-e; Additional file 5: Movie S5). Rod-like subcellular structures (which may represent mitochondria [43]; see below), and to a lesser extent, their surroundings, were lit sequentially, a few cells at a time (Figure 2c-e). The velocities of the Ca^2+^ waves appeared to decrease as they propagated, but their intensities varied in different cells (Figure 2e).

We examined the relationship between the travel distance of a wave and its velocity (Figure 2f). The velocities of the Ca^2+^ waves were 1–10 μm/sec, and the maximum traveling distance was approximately 1 mm. A reasonable correlation value of *r* = 0.67 (Pearson; *p* = 8.3 × 10^−6^) was obtained. The waves that traveled long distances were faster than those that traveled short distances. The propagation of these Ca^2+^ waves could be described as negative acceleration with a varied initial velocity.

**Figure 3:**
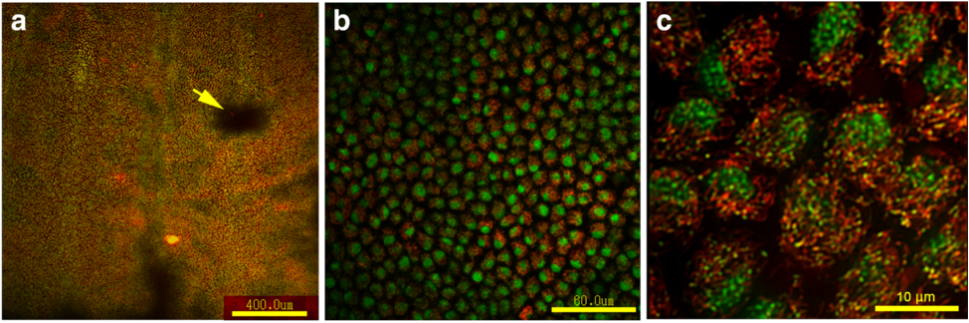
**Pupal wing epithelial cells double-stained with SYBR Green I for nuclei and MitoTracker Orange for mitochondria. (a)** The border region containing the M_3_ and CuA_1_ compartments. The prospective eyespot area is indicated by an arrow. The scale bar represents 400 μm. **(b)** High magnification. The scale bar represents 60 μm. **(c)** Further higher magnification. The cellular diameter is approximately 10 μm. Many mitochondria are observed as rod-like structures. The yellow dots are mitochondrial DNA. The scale bar represents 10 μm. Similar results have been previously published [43].

Double-staining with SYBR Green I for nuclei and MitoTracker Orange for mitochondria revealed epithelial cells in the pupal wing tissues (*n* = 7) (Figure 3). The mitochondrial staining pattern (with many rod-like structures inside of a cell) that was revealed by MitoTracker Orange was similar to the staining pattern of the Ca^2+^ indicators at the cellular level, as shown in Figure 2c, d.

### Damage-associated production of Ca^2+^ waves

**Figure 4:**
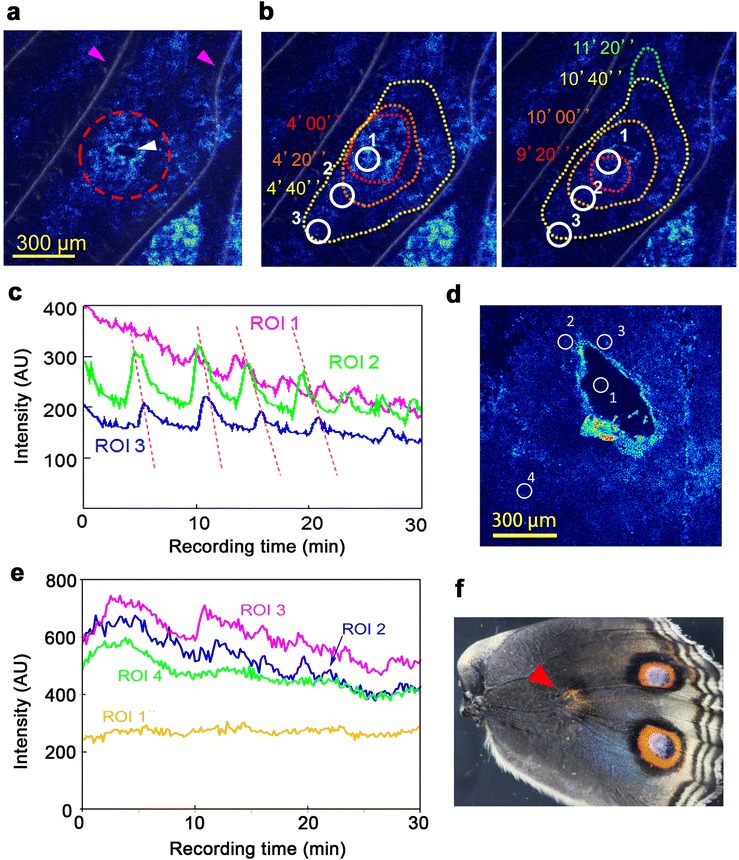
**Damage-associated Ca** ^**2+**^ **waves. (a)** Damage-associated propagating waves. The damaged site is indicated by a white arrowhead and a broken red circle. The pink arrowheads indicate the wing veins. The scale bar represents 300 μm. **(b)** The circular propagation of wave fronts from the damaged site shown in **a** from 4’00” to 4’40” (left panel) and from 9’20” to 11’20” (right panel). Three ROIs were set for analysis of intensity changes. See also Additional file 6: Movie S6. Damage-associated waves were induced successfully in 3 out of 9 treated individuals. **(c)** Fluorescence intensity changes over time in the 3 ROIs set in **b**. The identical waves are indicated by dashed lines. **(d)** Damaged wings with large elevations in Ca^2+^ signals around the damaged sites were observed in 6 out of 9 treated individuals. No generation of clear propagating Ca^2+^ waves was observed, but there were many small flickering waves around the damaged sites. ROIs were set for analysis of intensity changes. The scale bar represents 300 μm. **(e)** Fluorescence intensity changes over time in the 3 ROIs set in **d**. From ROIs 2 and 3, which were located at the periphery of the damaged site, non-expanding Ca^2+^ oscillations were observed over time. **(f)** A successfully damage-induced ectopic color pattern. This wing is not from the same individual that showed the Ca^2+^ signals in **a**-**e**. Similar results in the same species have been previously published [40].

Because physical damage is known to induce small ectopic eyespots in the hindwings of this species of butterfly [40], an intact hindwing was damaged by the tip of a needle in a background area of the basal region, producing a hole with a diameter of approximately 100–300 μm (Figure 4a). In 3 of the 9 individuals treated, we observed both an increase in fluorescence intensity around the damaged site and clear expanding Ca^2+^ waves radiating from the damaged site (Figure 4b; Additional file 6: Movie S6) at intervals of approximately 5 min (Figure 4c). The expanding ring waves at a given site did not arise spontaneously from undamaged areas and were similar to those radiating from the immediate periphery of the prospective eyespot. Indeed, in the remaining 6 cases treated, we also observed large increases in fluorescence intensity that changed over time without expanding movement immediately around the damaged site (Figure 4d, e; Additional file 7: Movie S7). In addition to the elevated Ca^2+^ signal immediately around the damaged site, flickering intensity changes in small areas around the site were observed (Figure 4d, e; Additional file 7: Movie S7).

We confirmed the previous finding [40] that an ectopic pattern was induced in the adult hindwings after damage (*n* = 2) in this species (Figure 4f), although its induction in the adult wings required more severe damage than the induction of Ca^2+^ waves (see Methods). The induced ectopic pattern was composed of an outer black ring, middle orange ring, and bluish focal area, similar to a normal eyespot (Figure 4f).

### Pharmacological modification of Ca^2+^ waves

**Figure 5:**
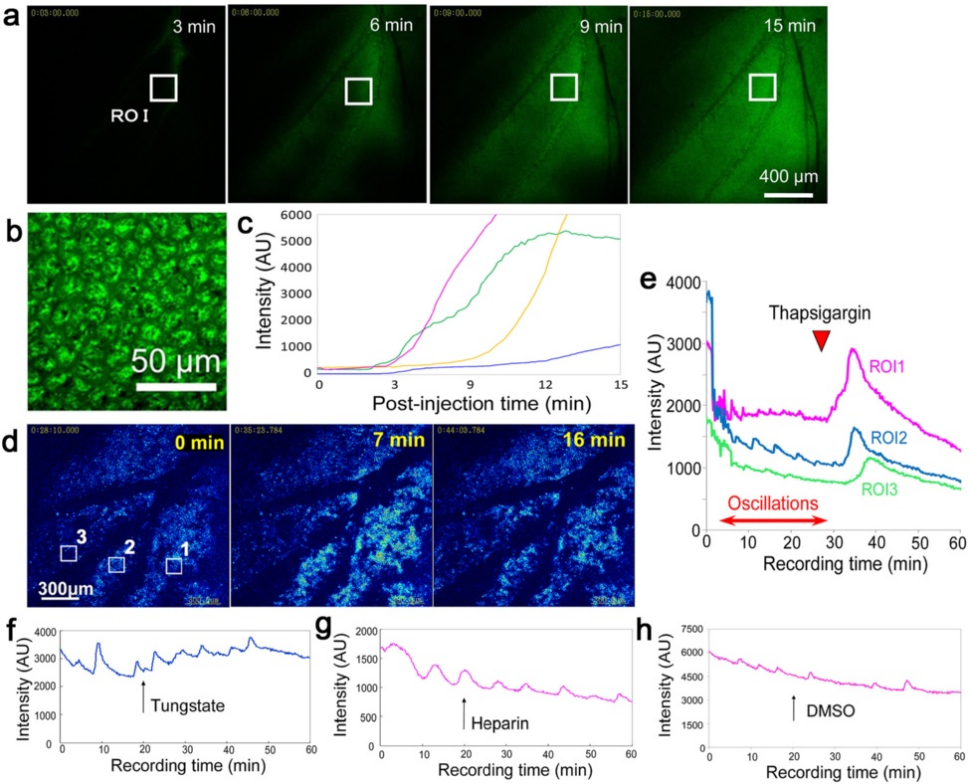
**Thapsigargin-induced changes in Ca** ^**2+**^ **signals. (a)** Time course of the appearance of rhodamine 123 in the wing tissues after injection into the abdomen. A single ROI was set for quantitation of intensity changes. The scale bar represents 400 μm. **(b)** High magnification of the ROI indicated in **a** at 15 min. Epithelial cells are identified. The scale bar represents 50 μm. **(c)** Intensity changes over time in the ROI. The changes shown in **a** are graphically displayed by the pink curve. The other curves of different colors were recorded from different individuals in the same manner. **(d)** An area of the hindwing around the prospective discal spot (i.e., the basal region). Three ROIs were defined for analysis, which are depicted in **e**. Thapsigargin was applied at 0 min. At approximately 7 min after injection of thapsigargin, intracellular Ca^2+^ increased dramatically. The scale bar represents 300 μm. See also Additional file 7: Movie S7. **(e)** Fluorescence changes over time. The oscillations before the injection of thapsigargin were abolished, and the Ca^2+^ signals increased suddenly. Thapsigargin injection point is indicated by an arrowhead. **(f-h)** Tungstate, heparin, and dimethyl sulfoxide (DMSO) injections resulted in no changes in oscillations and Ca^2+^ levels. Injection point is indicated by an arrow in each panel.

Some chemicals have been reported to induce color pattern modifications when injected immediately after pupation. The first chemical modifier that was discovered for the injection of chemicals into pupae was sodium tungstate [33], and heparin and other related chemicals were subsequently found to induce similar modifications [36]. Additionally, thapsigargin, which is a chemical that affects the intracellular trafficking of Ca^2+^, has been shown to increase dark-colored scales in this species [35]. To examine the possible effects of these chemicals on spontaneous Ca^2+^ waves in real time, we first examined the length of time required for an injected dye to reach wing tissue. Rhodamine 123 injected into the abdomen was detected in the wing tissue at 3–10 min after injection (*n* = 4) (Figure 5a-c).

We defined 3 ROIs in the region of the wing tissue at which the prospective discal spot was located (i.e., the basal region) (Figure 5d). After we confirmed the recording of spontaneous Ca^2+^ waves in the visual field in addition to the ROIs, thapsigargin was injected into the abdomen, which caused a transient increase in the intracellular Ca^2+^ concentration and the disappearance of spontaneous waves at 3–10 min after injection (*n* = 4) (Figure 5d, e; Additional file 8: Movie S8). Injections of tungstate (*n* = 7), heparin (*n* = 5), and dimethyl sulfoxide (DMSO) (*n* = 3) did not cause any changes in the spontaneous Ca^2+^ waves (Figure 5f-h).

**Figure 6:**
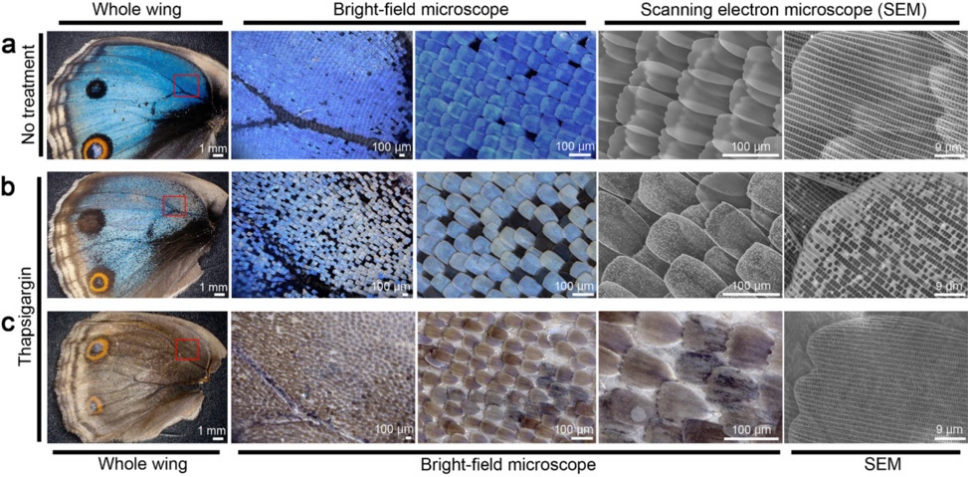
**Scale structures of hindwings of adults treated with thapsigargin at the pupal stage.** The boxed areas of 3 individual wings shown on the left side were examined by bright-field microscopy and scanning electron microscopy. **(a)** A normal wing. **(b)** A thapsigargin-treated wing. There are a lower number of cover scales (blue scales), resulting in the exposure of many ground scales (black scales). The cover scales themselves are dull in color, which may be because of the abnormal ridges of the fine-scale structures (right-most panel). **(c)** Another thapsigargin-treated wing. This wing is severely affected, with almost no cover scales. The ground scales are also dull in color, and the dark pigment appears to be distributed unevenly. The ridges of the fine-scale structures appear normal (right-most panel).

We confirmed that thapsigargin (*n* = 13) (Figure 6a-c), tungstate (*n* = 2) (data not shown), and heparin (*n* = 7) (data not shown) induced characteristic color pattern changes in the adult wings when injected into the abdomen. In contrast with normal wings, the thapsigargin-treated wings showed incomplete cover scale development (7 individuals, 54%) in 13 individuals (Figure 6b, c). Furthermore, increases in dark-colored scales and fuzzy boundaries of color pattern elements were observed in 7 individuals (54%) in each category (data not shown; refer to Otaki et al. [35]). In addition, the arrangement and fine structures of the scales appeared to be disturbed, as shown by the scanning electron microscope images and the dull, blue scale color (Figure 6b, c).

## Discussion

Using a unique technique for imaging developing butterfly wings, we discovered spontaneous intercellular Ca^2+^ waves that propagated slowly across pupal wings. Our experimental system allowed for the non-invasive observation of developing wings *in vivo*, and the individual pupae used for imaging normally developed the full adult color patterns [40,42,43], excluding the potential artifacts that may be associated with the experimental system. Interestingly, Ca^2+^ waves were not detected in larval wings *in vivo* (*n* = 10), which suggests that they play developmental roles only in pupal wings.

For many waves, we were unable to find a clear point of origin or trajectory. We believe that this was largely because our visual field was not large enough to find originating points for the waves. Additionally, wave interactions complicated this issue. Variability in waves may be due to technical reasons, such as inherent individual variation, slightly different locations, the timing of recordings, uneven loading of the indicator from individual to individual and from location to location, and the level of bleaching of the indicator (see Methods). It is worth noting that even the timing of gene expression in butterfly wings varies within a population during development [44].

In contrast, some waves clearly originated from the immediate periphery of the prospective eyespot (see Figure 1f-h), the prospective discal spot (see Figure 1i-l), and the damaged site (see Figure 4a-c). Wave patterns appeared to differ between the two regions examined (the border and basal regions) (see Figure 1f-h for the border region and Figure 1i-l for the basal region). These results suggest an involvement of Ca^2+^ waves in wing development. Physical damage (see Figure 4) and pharmacological experiments (see Figure 5) also support this interpretation. However, we admit that the present study is mainly descriptive, and a direct functional role of Ca^2+^ waves cannot be rigorously proven by these methods because many other molecular pathways may be modified by physical damage and chemical injections, which could then indirectly change scale morphology and color patterns in the long term.

Nonetheless, because many other studies have demonstrated instructive roles of Ca^2+^ waves in developmental systems [4,7,9,12,13], we believe that the butterfly wing system is not an outlier. Indeed, Ca^2+^ waves travel long distances in the liver and are terminated by annihilation collision with another wave front [10,11], similar to our observations in butterfly wings. In zebrafish, spontaneous Ca^2+^ waves coordinate cellular processes, including morphogenesis during development [12,13]. The waves appear to be generated from cells of morphological importance in the developing neocortex, such as S-phase cells [4].

**Figure 7:**
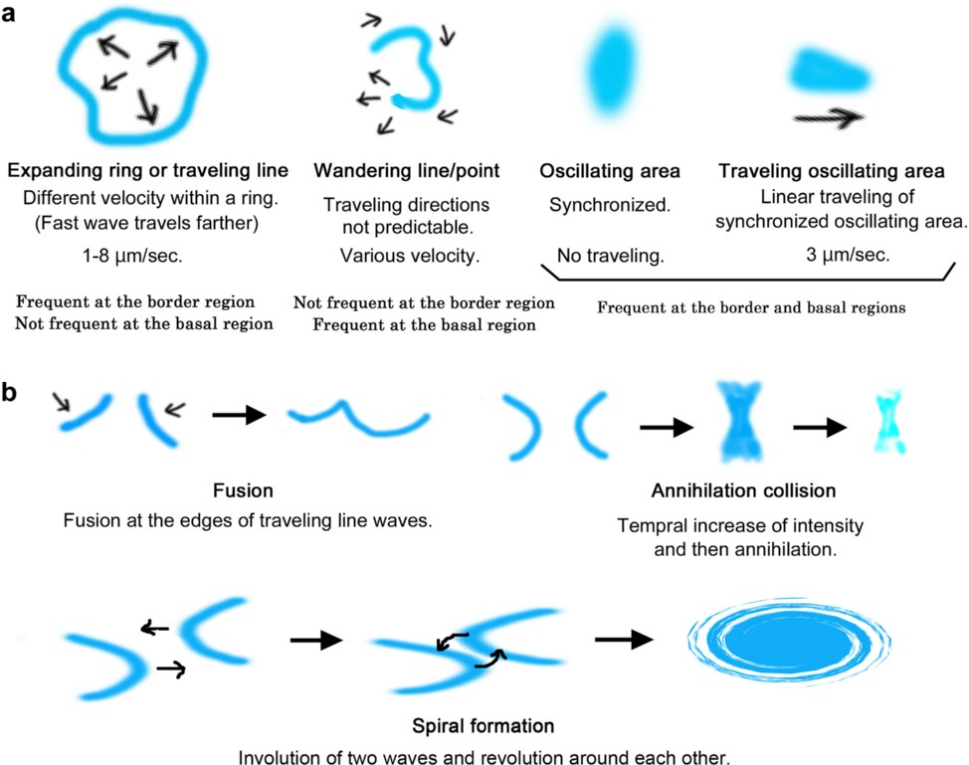
**Behavior of Ca** ^**2+**^ **waves. (a)** Four patterns of Ca^2+^ waves and oscillations. Notable features, velocity, and an estimated frequency of occurrence are indicated. **(b)** Three patterns of wave interactions, fusion, annihilation collision, and spiral formation.

Some specific aspects of butterfly Ca^2+^ waves are noteworthy. First, the interactions between two or more Ca^2+^ waves are diverse, but may have some general rules (Figure 7). For example, two progressive waves may fuse, but they may also collide with each other and decrease in intensity. In addition, two waves may occasionally form a spiral pattern around each other.

Second, the frequency of individual waves also varied, but it was typically between 1 and 4 cycles in 10 min, roughly corresponding to 2 to 7 mHz. To detect this type of low-frequency wave, long-term real-time recordings are required, which were achieved with our system.

Third, these waves are long-range. The maximum travel distance that we observed was approximately 1 mm, which is an underestimate because a greater distance is expected if the visual field under the microscope is wider or if the detection sensitivity of the system is higher. Nonetheless, this 1-mm distance was exceptionally long, spanning at least 50–100 cells, given that a single epithelial cell is approximately 10–20 μm in diameter in pupal wing tissue (see Figure 3) [43]. These findings suggest that approximately 10^4^ cells are involved in wave-generating activity when a wave propagates radially from a designated point in a two-dimensional plane (based on a simple calculation, *area* = *πr*^2^). It is possible that a long-range wave may reach every single cell of a given wing surface, which may occasionally result in the wing-wide synchronization of waves.

Fourth, Ca^2+^ waves in the wings are slow, traveling at 1–10 μm/sec (see Figure 2f). The wing waves that we detected could be categorized as slow waves (in contrast to fast waves) [45,46], which have been hypothesized to be caused by stretch-sensitive channels that allow extracellular Ca^2+^ flow into the cell [45,46]. This activity can occur in developing epithelial cells of butterfly wing tissues because each cell is conical in structure with relatively long dendritic processes; thus, the deep sides are likely to be physically flexible [43]. A more detailed morphological characterization of epithelial cells is currently underway in our laboratory.

Fifth, the velocity of Ca^2+^ waves appears to be distance-dependent (see Figure 2f), indicating that the initial velocity may determine the traveling distance. The waves appear to travel in a slightly decelerating motion. The velocity that was recorded in this study is similar to that of waves that have been detected in slime mold, in which they may be used for cellular integration [14].

Interestingly, Ca^2+^ waves can be initiated by physical damage (see Figure 4), which is reminiscent of those that have been induced in damaged fish skin [47], cultured rat skin [48], and damaged brain tissue [49]. It is therefore possible that damage-associated Ca^2+^ waves play a role in producing ectopic eyespots.

The biological functions of Ca^2+^ waves have also been assessed by pharmacological experiments (see Figures 5, 6). Neither tungstate nor heparin, which are two known color-pattern modifiers, affects any aspect of Ca^2+^ waves. DMSO, which is used as a solvent of thapsigargin, also does not cause any change in these waves. In contrast, thapsigargin, which modifies background color [35], transiently increases the intracellular Ca^2+^ concentration and abolishes these waves, indicating that it functions as a Ca^2+^-ATPase inhibitor in the endoplasmic reticulum (ER) membrane [50,51]. These short-term effects of thapsigargin, which were expected because of its well-known function, were observed immediately after injection, confirming its successful induction of ER stress. Because the specific effects of thapsigargin have been well established [50,51], we believe that this short-term effect is caused by thapsigargin directly, and not by indirectly through unknown side effects.

The long-term effects of thapsigargin on scales in this species are known [35], and similar effects have also been reported following ionomycin injection [35]. These pharmacological agents both work through elevating the cytosolic calcium concentration. Therefore, it has been speculated that calcium may play a role in scale development [35]. In the present study, in addition to cover scale color, we demonstrated that thapsigargin also affected the arrangement, shape, microstructure, and formation of cover scales. It is unclear whether the short-term effects of thapsigargin eventually led to long-term phenotypic effects without any side effects. In other words, we do not know whether its effects on cover scales are direct or indirect results of the abolishment of Ca^2+^ waves. However, we speculate that long-range, slow Ca^2+^ waves may play important roles in multiple aspects of normal cover scale development. The disorder of the scales suggests that Ca^2+^ waves may function as synchronization signals for cell growth and division, resulting in wing-wide phenotypic coordination [40,41]. Alternatively, waves may be used for interactions between elements. It has been reported that the size and morphology of elements are affected by nearby elements [29,42]. To demonstrate a direct functional role of Ca^2+^ waves in wing development, genetic manipulations may be required in the future. A relatively easy-to-use method is baculovirus-mediate gene transfer, which can be successfully used to deliver a gene into the wings of this butterfly species [52].

The mechanism that underlies the intercellular propagation of Ca^2+^ waves is currently unknown. Inside a cell, mitochondria appear to play a similar role as in cochlear supporting cells [53]. As we could not detect any changes in color patterns or Ca^2+^ waves when octanol, which is a general gap-junctional inhibitor, was injected (data not shown), signaling may be mediated by a chemical (such as ATP) and a receptor via the extracellular space. The Ca^2+^ waves detected in this study are somewhat similar to progressive waves from an organizing center that have been predicted by the induction model for positional information [30,31]. However, the morphogenic signal that is predicted by the induction model consists of ultra-slow waves that are more likely to be gene expression waves [54,55] or other similar waves. The relationship between Ca^2+^ waves and the predicted ultra-slow waves remains to be clarified. For example, Wnt ligand, which may be involved in color-pattern formation [56,57], may be induced by Ca^2+^ waves.

## Conclusions

This study detected, for the first time, spontaneously produced Ca^2+^ waves that travel slowly over long distances during butterfly wing development. These findings were made possible by our technological advancements in butterfly wing live imaging. Moreover, Ca^2+^ waves were generated by physical damage, which is known to produce ectopic eyespots. Thapsigargin injection abolished the Ca^2+^ waves and resulted in abnormal scales and patterns. These results suggest that spontaneous Ca^2+^ waves play critical roles in scale development and color pattern formation, which are involved in two-dimensional morphogenesis in butterfly wings. The discovery of long-range, slow Ca^2+^ waves that may contribute to pattern formation is a significant advancement in the developmental biology of pattern formation [58,59] and in signaling biology [60].

## Methods

### Butterfly rearing

Throughout this study, we used the blue pansy butterfly *J. orithya* (Linnaeus, 1758) (see Figure 1a). No specific permissions were required to collect this species in Okinawa, where this study was conducted. This species is not endangered or protected and is one of the most common butterflies in Okinawa. Adult females and larvae were collected from Ishigaki-jima Island or Okinawa-jima Island in the Ryukyu Archipelago, Japan, and eggs were collected from these females. The larvae were fed natural host plants and maintained under a 15L-9D cycle at approximately 27-28°C.

### Operations and loading

To load a Ca^2+^ indicator into wing tissue, the left pupal forewing was curled up from the apical site using forceps within 45 min after pupation, as previously described [40,42,43]. This operation is possible because the pupal cuticle is still soft immediately after pupation. It is important to note that this operation simply involves the displacement of the hindwing; thus, it is non-invasive. Left intact, an operated pupa can develop normal color patterns and eclose [40,43]. We also confirmed that displaced floating wings (not placed on a piece of glass) in modified Ringer’s solution (see below) exhibited Ca^2+^ waves (data not shown). Therefore, the *in vivo* images that we obtained in the present study can be considered to be free from artifacts. The hindwing epithelial cells were loaded with modified low-pH Ringer’s solution with the following composition: NaCl (183 mM), KCl (20 mM), CaCl_2_ (5.6 mM), MgCl_2_ (0.83 mM), and citric acid (0.19 mM) containing Fluo-4 AM (Fluo-4 acetoxymethyl ester; Molecular Probes, Eugene, OR, USA) or Fluo-8 AM (TEFLabs, Austin, TX, USA) at a final concentration of 20 μM with brief sonication. We found that the addition of a small amount of citric acid to the loading solution increased the efficiency of Fluo-4 loading into the wing tissue. This addition was not necessary when using Fluo-8. Approximately 50 μL of loading solution was sandwiched between the fore- and hindwings for 60 min at approximately 27°C under high-humidity conditions to avoid the evaporation of the liquid (see Figure 1c). This sandwich method has been previously employed to load various chemicals into pupal wing tissues [42]. The loading solution was then washed out thoroughly before recording.

### Calcium imaging

Naked hindwings were placed on cover glasses (see Figure 1d) and observed using an inverted microscope after a 10-min resting period. We focused on two areas (the basal and border regions) on the hindwing (see Figure 1b, e). The laser output at 488 nm was set at 6 mW, the electron-multiplying (EM) gain was set to 255 (maximum), and the exposure time was set to 300 ms at a defined interval of 10 sec over a 60-min period. This protocol resulted in 361 images, which were then compiled to generate a movie. Images of calcium changes were analyzed using pseudocolors. The real-time confocal microscope imaging system used in this study included an Eclipse Ti-U inverted epifluorescence microscope (Nikon, Tokyo, Japan), a CSU-X1 laser-scanning unit with a 520/25-nm band-pass filter (Yokogawa, Tokyo, Japan), and an ImagEM EM-CCD camera (Hamamatsu Photonics, Hamamatsu, Japan). An AQUACOSMOS/RATIO calcium-ion analysis system (Hamamatsu photonics, Hamamatsu, Japan) was used as system control software. Autofluorescence from the tissues without a loaded indicator was found to be minimal or null. The recorded signal intensity in arbitrary units (AUs) varied from individual to individual. This finding likely occurred due to the following 5 reasons: (1) the fluorescence indicator may have gradually been bleached; (2) the level of fluorescence indicator in the cells may have varied due to uneven loading efficiency from individual to individual and from location to location; (3) the levels of Ca^2+^ waves themselves may have inherently varied among individuals and also among locations; (4) the operation itself is simple, but an unsuccessful operation may have occurred, mainly due to a leakage of hemolymph, which may have weakened the pupae; and (5) laser-based observations over a long period of time may have damaged the pupae.

### Nuclear and mitochondrial staining

We employed SYBR Green I (Life Technologies, Carlsbad, CA, USA) and MitoTracker Orange (Life Technologies) to stain the nucleus and mitochondria, respectively. For SYBR Green I, the original solution from the manufacturer was diluted 2,000 fold in the final loading solution, which was used for the staining process. For MitoTracker Orange, the concentration of the final loading solution was 100 μM. The solutions were loaded similarly as the Ca^2+^ indicators. The same confocal microscope system described above was used. Confocal images were obtained by making optical slices with 0.2-μm steps of 2.0 μm in thickness. The excitation and emission wavelengths for the images were 488 nm and 520/25 nm for SYBR Green I and 561 nm and 617/73 for MitoTracker Orange, respectively. Essentially similar results have been previously reported [43].

### Velocity and distance analysis

The acquired serial images of Ca^2+^ waves were examined with AQUACOSMOS 2.6 software. We focused on the waves that were relatively easily identifiable during propagation. The locations and times of the wave fronts were monitored manually using serial images. We focused on 5 waves that were not mutually overlapping from a single tissue. We examined 7 individuals, resulting in the assessment of a total of 35 waves.

### Damage experiments

After the loading solution was washed out, the pupal hindwings were physically damaged using an insect pin (Shiga Konchu, Tokyo, Japan; 110 μm in diameter at the columnar body and a few micrometers at the tip) in a background area of the basal region to induce Ca^2+^ waves. Physical holes were created on the surfaces of the early pupal hindwings without hemolymph leakage. This minor damage did not induce ectopic spots on the adult wings. To induce ectopic color patterns, a large insect pin (Shiga Konchu; 350 μm in diameter at the columnar body and a few micrometers at the tip) was required. Indeed, small areas of damage were able to heal quickly without leading to a color pattern change.

### Chemical injections

To examine whether Ca^2+^ waves respond to chemicals, pupae were injected with 2.0 μL of a solution containing the following chemicals into the abdomen on the opposite side of the operated hindwing after indicator loading: sodium tungstate (1.0 M in ddH_2_O), thapsigargin (10 mM in dimethyl sulfoxide for calcium imaging of pupae because treated individuals did not have to survive to the adult stage, and 1 mM in dimethyl sulfoxide for scale modifications in adults because treated individuals had to survive to the adult stage), and heparin (5.0 U/mL in dimethyl sulfoxide) using an Ito microsyringe (Fuji, Shizuoka, Japan). The exact mechanisms of action of tungstate and heparin in butterflies are unknown, but thapsigargin is a well-known inhibitor of Ca^2+^-ATPase in the ER membrane that increases the cytosolic Ca^2+^ concentration [50,51]. In addition, 1.0 M NaCl (*n* = 4), ddH_2_O (*n* = 3), and dimethyl sulfoxide (*n* = 3) were injected as controls, resulting in no changes in pupal Ca^2+^ waves or adult color patterns. It has been shown that no color pattern modifications in adult wings are induced by these chemicals [33,35]. Detection of chemicals in the wing tissue was tested by the injection of rhodamine 123 (1 mM in dimethyl sulfoxide). We reported the number of individuals whose adult wings were affected by the chemicals and not the number of individuals injected because the sensitivities of the butterflies to the chemicals were highly variable.

### Scale images

Scale images were taken using a high-resolution high-depth Keyence VHX-1000 digital microscope (Osaka, Japan) and a Hitachi High-Tech TM3030 tabletop microscope, which is a benchtop-type scanning electron microscope (Tokyo, Japan).

## Additional files

Additional file 1: Movie S1. Spontaneous Ca^2+^ waves from the prospective eyespot area in the M_3_ and CuA_1_ compartments (the border region). See Figure 1f-h for details. Additional file 2: Movie S2. Spontaneous Ca^2+^ waves around the prospective discal spot (the basal region). See Figure 1i-l for details. Additional file 3: Movie S3. Complex movement of spontaneous Ca^2+^ waves around the prospective discal spot (the basal region). A spiral wave as an interaction between two facing waves is observed. Additional file 4: Movie S4. Ca^2+^ waves traveling diagonally across the visual field. This type of non-complex wave was used to measure traveling distance and velocity. See Figure 2a-b for details. Additional file 5: Movie S5. Ca^2+^ waves traveling at the cellular level. The Ca^2+^ signal moves a few cells at a time. See Figure 2c-e for details. Additional file 6: Movie S6. Damage-associated Ca^2+^ waves. Expanding ring waves radiating from the damaged site are observed. See Figure 4a-c for details. Additional file 7: Movie S7. Damage-associated Ca^2+^ waves. Flickering waves around the damaged site are observed. See Figure 4d, e for details. Additional file 8: Movie S8. Response of Ca^2+^ waves to thapsigargin injection. Upon injection, the Ca^2+^ level is elevated, as expected from the pharmacological activity of thapsigargin. See Figure 5d, e for details.
